# A hydrotrope pretreatment for stabilized lignin extraction and high titer ethanol production

**DOI:** 10.1186/s40643-022-00530-6

**Published:** 2022-04-04

**Authors:** Hairui Ji, Le Wang, Furong Tao, Zhipeng Yao, Xuezhi Li, Cuihua Dong, Zhiqiang Pang

**Affiliations:** 1 State Key Laboratory of Biobased Material and Green Papermaking, Qilu University of Technology (Shandong Academy of Sciences), 3501 Daxue road, Jinan, 250353 China; 2 School of Chemistry and Chemical Engineering, Qilu University of Technology (Shandong Academy of Sciences), 3501 Daxue road, Jinan, 250353 China; 3 State Key Laboratory of Microbial Technology, Shandong University, Jinan, China

**Keywords:** Pretreatment, Recyclable hydrotrope, Lignin quenching agent, Q-SSF, Ethanol

## Abstract

The biomass pretreatment strategies using organic acids facilitate lignin removal and enhance the enzymatic digestion of cellulose. However, lignin always suffers a severe and irreversible condensation. The newly generated C–C bonds dramatically affect its further upgrading. In this study, we used a recyclable hydrotrope (*p*-Toluenessulfonic acid, *p*-TsOH) to dissolve lignin under mild condition and stabilized lignin with a quenching agent (formaldehyde, FA) during extraction, achieving both value-added lignin extraction and efficient enzymatic saccharification of cellulose. Approximately 63.7% of lignin was dissolved by 80% (wt. %) *p*-TsOH with 1.5% FA addition at 80 °C, 30 min. The obtained lignin was characterized by FTIR spectroscopy, TGA, 2D HSQC NMR spectroscopy, and GPC. The results indicated that the extracted lignin exhibited excellent properties, such as light color, a low molecular weight (*M*w, 5371 g/mol), and a narrow polydispersity (*M*_w_/*M*_n_, 1.63). The pretreated substrate was converted to ethanol via a quasi-simultaneous saccharification and fermentation process (Q-SSF). After fermentation of 60 h, the ethanol concentration reached 38.7 ± 3.3 g/L which was equivalent to a theoretical ethanol yield of 82.9 ± 2.2% based on the glucan content, while the residual glucose concentration was only 4.69 ± 1.4 g/L. In short, this pretreatment strategy protected lignin to form new C–C linkages and improved the enzymatic saccharification of glucan for high-titer ethanol production.

**Graphical Abstract:**

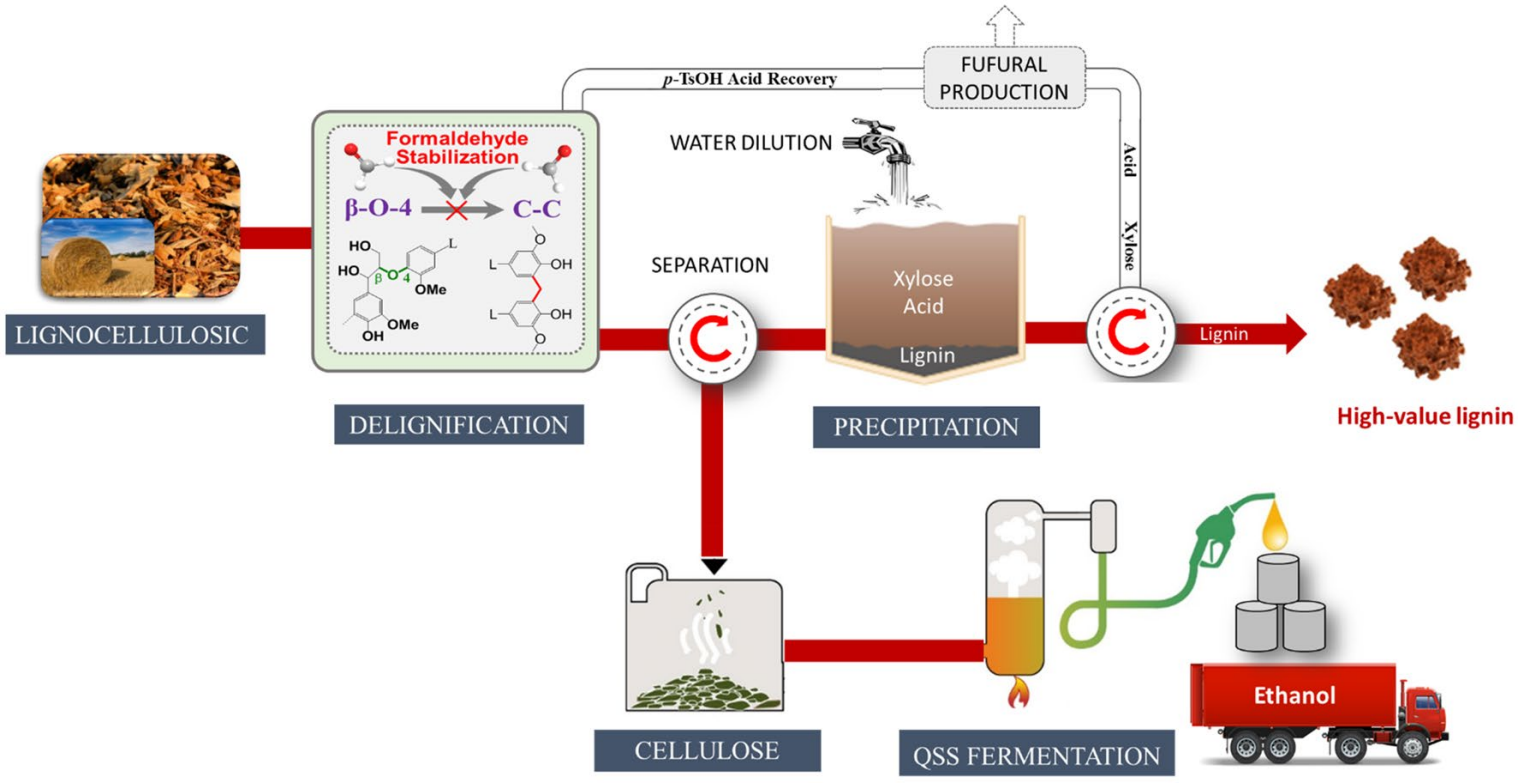

## Introduction

The non-renewability and limited reserves of fossil resource have stimulated a worldwide initiative to find a sustainable resource for production of fuel and chemicals (Khan et al. 2011). Lignocellulosic biomass, the most abundant renewable feedstock on the earth, has received considerable attention as a potential alternative of petroleum in recent years. Lignocellulose is mainly composed of cellulose, hemicellulose, and lignin along with small amounts of pectin, protein, extractives, and ash. The enzymatic hydrolysis of cellulose and hemicellulose can release various monosaccharides, good precursors for the production of fuels and value-added chemicals. However, the three polymers (cellulose, hemicellulose, and lignin) linked into a complex matrix forming a protective barrier against enzyme and microbial degradation (Yi et al. 2014). Therefore, a pretreatment process is essentially required to facilitate the enzymatic accessibility of carbohydrate components (cellulose and hemicellulose) and promote the conversion efficiency of lignocellulosic biomass. Current pretreatment techniques involved physical, physical–chemical, chemical, biological or combined methods (Kumar et al. 2009; Lynd et al. 2022). Physical pretreatments include grinding, extrusion (Zheng and Rehmann 2014), and irradiation (ultrasound and microwave) (Gao et al. 2021). Physical–chemical pretreatments typically conclude hydrothermolysis (liquid hot water) (Negro et al. 2003; Zhou et al. 2016), steam explosion (Singh et al. 2015), ammonia fiber explosion (AFEX) (Murnen et al. 2007), and CO_2_ explosion (Kim and Hong 2001). Chemical pretreatments refer to using dilute acids (Jung et al. 2013), alkalis (NaOH, Ca(OH)_2_, KOH, and NH_3_.H_2_O), organosolv (Zhao et al. 2009), and ionic liquids (Tadesse and Luque 2011; Zhang et al. 2021) to alter the physical and chemical structure of lignocellulosic biomass. Although these pretreatment methods can effectively break down the structural barrier from lignin, most of them require special reaction equipment and huge energy consumption. Compared with physical and chemical methods, biological pretreatments have many advantages, such as low energy consumption, no chemicals addition, and mild reaction condition (Yi et al. 2014). However, long pretreatment time has limited its utilization on commercial scale (Taherzadeh and Karimi 2008). In addition, the effect of pretreatment methods on the changes in lignin molecular structure is generally ignored. Lignin, the second most abundant biopolymer on the earth comprising up to 20–35% of cell wall, is composed of three kinds of phenylpropane structural units, namely, guaiacyl (G), syringyl (S), and *p*-hydroxyphenyl (H) structures. These units are connected by carbon–carbon bonds and ether bonds (Costa et al. 2016). The aromatic and functional groups, such as methoxy, phenolic, alcoholic, carbonyl, and aldehyde groups, in lignin structure enable great potential applications in the preparation of biofuels, high-value chemicals, and composites. However, high pretreatment severity caused the skeleton rearrangement and condensation of lignin resulting in difficulties of depolymerize and further upgrading (Udeh and Erkurt 2016). Therefore, it is necessary to explore a pretreatment method that not only can improve enzymatic digestibility of glucan but also hinder the formation of C–C bonds in lignin molecules.

**Fig. 1:**
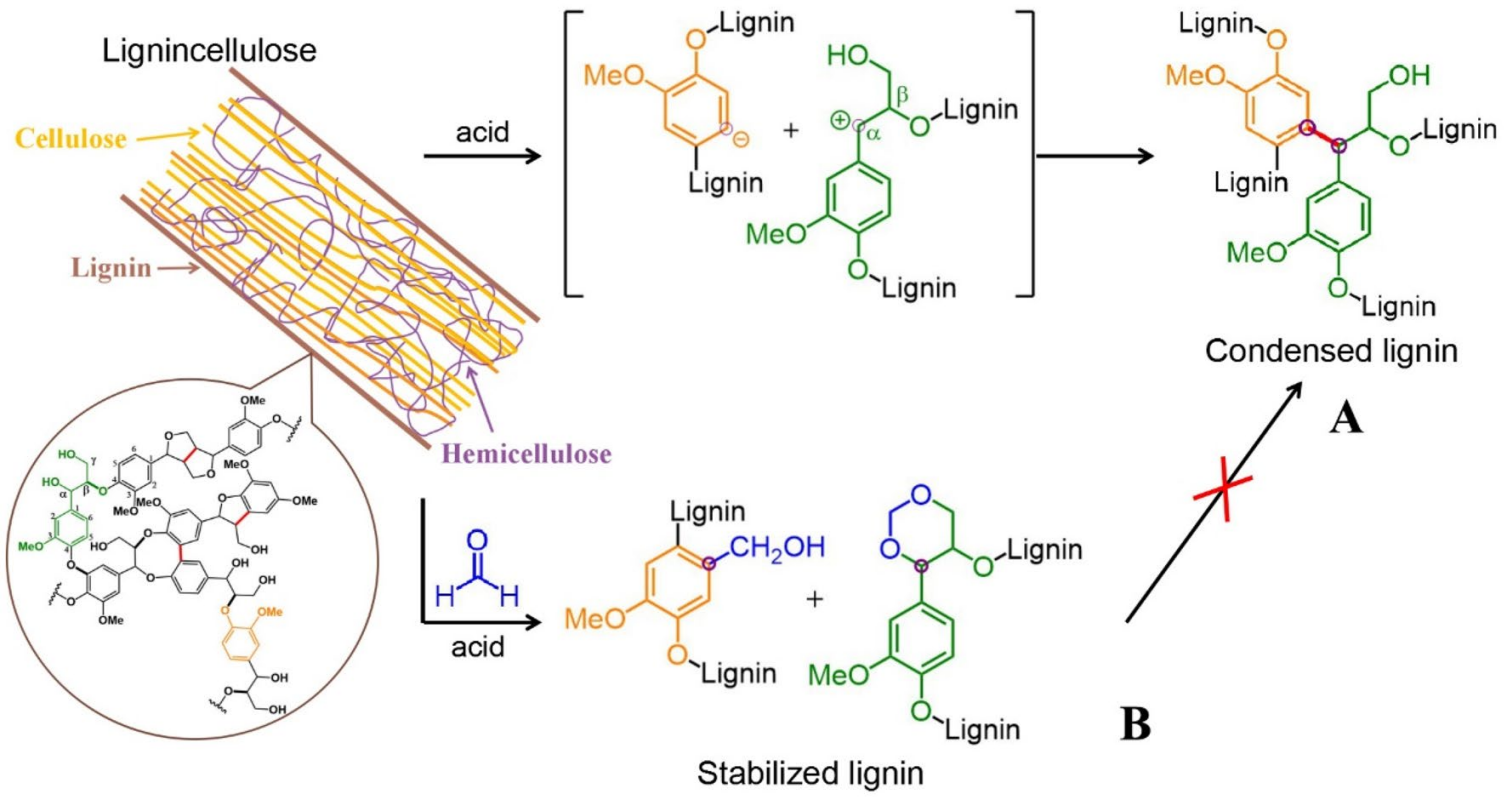
Condensation mechanism of lignin (**A**) and mechanism of formaldehyde preventing polymerization (**B**)

In previous studies, a recyclable acid hydrotrope (*p*-TsOH) has been mentioned for its unparalleled performance of delignification at mild temperatures below water boiling point. It is worth noting that 90% of lignin and hemicellulose were removed from *poplar* wood (Ni et al. 2017). By commercial crystallization technology, *p*-TsOH was recycled and reused for many times to achieve environmental sustainability (Ji et al. 2016). However, acid or high temperature conditions during lignin extraction caused malignant and irreversible condensation. The lignin condensation mechanism is shown in Fig. 1A. When lignin ether bonds are cleaved, the carbon cations in the alpha active sites of lignin side chain attack the negatively charged lignin aromatic rings to form stable carbon–carbon bonds (icon red key). A previous study has reported that using a protecting agent (FA) to stabilize lignin during extraction. FA molecule can quickly react with the α- and γ-hydroxyl groups of the side chain of lignin to form a stable 1, 3-dioxane structures through acetal reaction, thereby blocking the formation of benzylic carbocations. At the same time, FA can also react with the negatively charged benzene ring active sites to generate hydroxymethyl groups and deactivate the active sites preventing these positions from undergoing undesirable condensation reactions.

In this study, we used the hydrotrope *p*-TsOH to dissolve lignin and FA as quenching agent to stabilize lignin under mild conditions. An optimum pretreatment severity was determined according to the enzymatic hydrolysis of glucan and the lignin characterization results from FT-IR spectroscopy, TGA, 2D HSQC NMR spectroscopy, and GPC. Finally, the obtained pretreated substrate was converted into high-titer ethanol with a Q-SSF process. The novelty of the present study is to demonstrate a combination of high-efficiency dissolution of lignin by hydrotrope *p*-TsOH under mild conditions and avoidance of lignin molecules condensation using FA as quenching agent, achieving both value-added lignin extraction and efficient enzymatic saccharification of cellulose. Therefore, this study is important to the valorization of lignocellulosic biomass.

## Materials and methods

### Materials

*Poplar* wood chips were provided by Shan Dong Sun Paper Industry Joint Stock Co., Ltd. (Shandong, China), they were milled into particles with a size range of 40–60 mesh; *p*-TsOH and some chromatographic grade organic solvents were supplied by Macklin biochemical Co., Ltd. (Shanghai, China); FA solution (37–40%, wt.%) and H_2_SO_4_ (95–98%, wt.%) were purchased from Yantai Far Eastern Fine Chemical CO., Ltd. (Shandong, China); 1,4-dioxane was obtained from Kemiou Chemical Reagent CO., Ltd. (Tianjin, China); DMSO-*d*_6_ was provided by Cambridge Isotope Laboratories, Inc. (MA, United States). Cellulase (Cellic® CTec2) was supplied by Novozymes (Beijing, China).

### Pretreatment and lignin isolation

4 g wood powder with 40–60 mesh was added in 50 mL *p*-TsOH solution (60%, 70% and 80%). Reactions were conducted at temperatures of 70 °C and 80 °C with a reaction time range of 15 min, 30 min, and 60 min. Each pretreatment had 3 replications. At the end of each reaction, solid and spent liquor were separated using filter papers (15 cm, medium speed, Hangzhou Fuyang North Pulp paper CO., Ltd., Hangzhou, China). The solid was washed with pure water to neutral pH and freeze-dried for component analysis and enzymatic hydrolysis. The extracted lignin (EL) in filtrate was precipitated by adding water. After dialysis and freeze-drying, the purity of the obtained EL sample reached to 95.06% (wt.%). Besides, a control experiment with addition of 3% FA was performed to analyze the effects of FA molecule on blocking the formation of new C–C linkages. The EL and the cellulolytic enzyme lignin (CEL) (extracted from biomass by means of ball-milling and repeated enzymatic steps) were isolated and characterized for a comparison.

For the CEL extraction, the dried pretreated substrate was milled by a planetary ball mill for 4 h (400 rpm). The obtained powder was subjected to enzymatic hydrolysis (180 rpm, 50 °C, and 24 h) to decompose cellulose and hemicellulose. Subsequently, the lignin-rich solid was centrifuged (5000 rpm, 5 min), washed for three times, and freeze-dried. Finally, the lignin was extracted by dioxane/water (94:6, v/v), and the concentrated solution obtained by rotary evaporation was dropped into 20 ml of water to precipitate CEL with a purity of 92.58%.

The determination of specific surface area of raw material and pretreated substrate was performed on a mercury porosimeter (Autopore IV 9500, Micrometrics, USA) with stepwise pressure increment in the range from 0.0036 to 413 MPa. Before measurements, the pretreated wood chips were dried at 105 °C and subsequently degassed in a vacuum degree of 6.67 Pa at temperature of 20 °C. The surface morphological features were measured by a SEM apparatus (Hitachi S-3400 N, Japan) with an accelerating voltage of 5–10 kV.

### Enzymatic saccharification of cellulose

The enzymatic hydrolysis of cellulose was performed in a 250 mL conical flask with 50 mL citrate buffer (PH 4.8) at 50 °C with a rotate speed of 180 rpm. Cellulose-rich solid and cellulase (Cellic® CTec2) loadings were 2% and 15 FPU/g glucan, respectively. Samples were withdrawn (400 μL) at 3 h, 6 h, 12 h, 24 h, 36 h, 48 h, 60 h, and 72 h and centrifuged at 8000 rpm for 5 min. Glucose concentrations were determined by a biosensor analyzer (SBA-40E, Biological institute of Shandong academy sciences, Jinan, China). The calculation of the enzymatic saccharification of glucan was based on the following equation:

1 $$\mathrm{Yield} \left(\%\right)=\frac{{m}_{\mathrm{glucose}}}{{m}_{\mathrm{Raw}}\times {C}_{\mathrm{glucan}}/0.90}\times 100\%$$

where ${m}_{\mathrm{glucose}}$ is the total weight of produced glucose after enzymatic hydrolysis; ${m}_{\mathrm{Raw}}$ and ${C}_{\mathrm{glucan}}$ are the weight of used raw material (the pretreated substrate) and the content of glucan in raw material; 0.90 is a conversion coefficient of xylan to glucose.

### Lignin characterization

FT-IR spectra of lignin was recorded on a spectrophotometer (ALPHA, BRUKER, Germany) in a range from 500 to 4000 cm^−1^ at a scanning resolution of 4 cm^−1^.

Thermogravimetric analysis was performed on a thermogravimetric analyzer (TGAQ50, TA Instruments CO., Ltd., USA) according to a previous publication (Wen et al. 2013). The lignin samples were heated from 35 °C to 800 °C at an increment of 10 °C/min in nitrogen.

2D-HSQC NMR spectra were recorded on a Bruker 400 MHz spectrometer (AVANCEII, BRUKER, Germany) at 25 °C. Before measurement, 40 mg of lignin was dissolved in 0.6 mL of DMSO-*d*_6_.

Weight average molecular weight (*M*_w_), number average molecular weight (*M*_*n*_), and polydispersity index (*M*_w_/*M*_n_) were determined by a gel permeation chromatography (GPC) system (Waters CO., USA) with an ultraviolet detector. For lignin acetylation, 50 mg of dry lignin was dissolved in 4 mL solution of pyridine: acetic anhydride (1:1, v/v) and stirred in dark at 25 °C for 24 h. 20 mL acid water (pH 2) was dropped slowly into the concentrated solution to precipitate acetylated lignin. 20 μL acetylated lignin solution of 1 mg/mL was injected into a GPC system equipped a chromatography column (Styragel® HR 4 THF, 7.80 × 300 mm, Ireland). The operation was run at 35 °C using THF as eluent with at a flow rate of 0.6 mL/min. The GPC system was calibrated using standard polystyrene samples.

### Fermentation for ethanol production

The Q-SSF of cellulose was carried out in 150 mL erlrnmeyer flask with a solid loading of 15% and cellulose (Cellic® CTec2) addition of 15 FPU/g glucan. Enzymatic hydrolysis was carried out for 6 h at 50 °C and 180 rpm. Subsequently, the sample was inoculated with 2‰ active dry yeast (Angel Yeast Company, Hubei province, China) and incubated for 60 h. Then, the samples were withdrawn at 12 h intervals and detected the concentrations of sugar and ethanol on a HPLC system equipped with a refractive index detector (RID-20A) and a separated column (Aminex HPX-87H, Bio-Rad, CA, United States). All experiments were performed in triplicates.

## Results and discussion

### Pretreatment and enzymatic saccharification

**Fig. 2:**
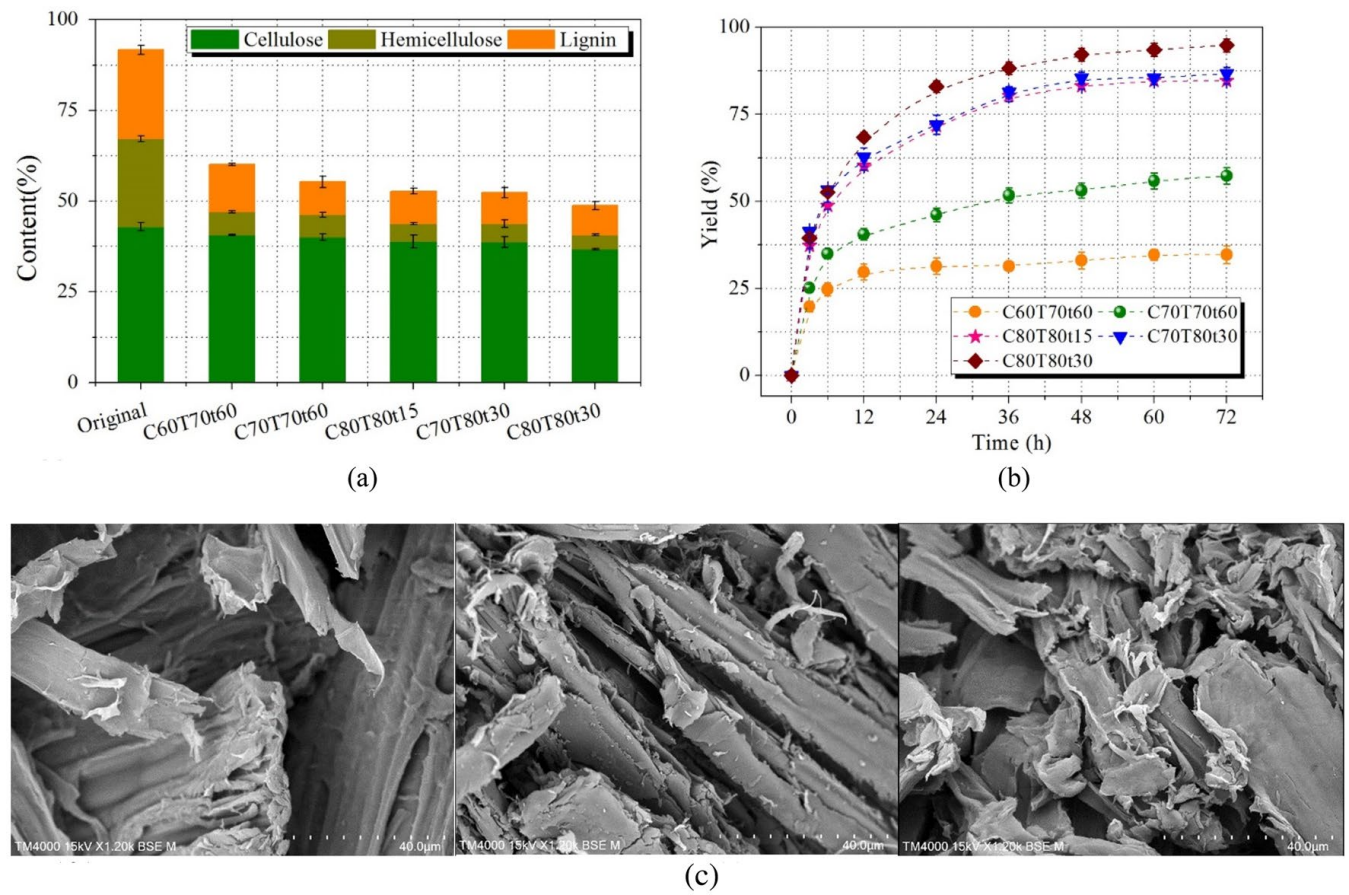
**a** Content of three components in the pretreated substrates (Cx stands for the acid concentration; Tx stands for the temperature; tx stands for the time), **b** Enzymatic saccharification of the pretreated substrates. **c** SEM images of raw material, the pretreated substrates obtained from C70T70t60 and C80T80t30

Figure 2 reveals the effect of different pretreatment conditions on the changes of components in biomass. Obviously, some of hemicellulose and lignin were removed after pretreatment leaving about 60% of original total content. The increased pretreatment severity significantly improved the dissolution of hemicellulose and lignin while caused negligible degradation of cellulose. The structural destruction will facilitate the contact between enzyme and cellulose, and can effectively promote the enzymatic digestibility of the substrate. Hence, an appropriate pretreatment intensity is necessary for biomass conversion. However, a high pretreatment severity inevitably caused chemical structure changes of lignin and reduced its potential application value. Therefore, a suitable pretreatment condition is essential to remove hemicellulose and lignin and maintain high-value utilization of lignin.

**Fig. 3:**
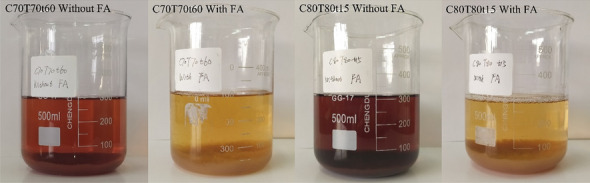
Morphology comparison of the extracted lignin with and without FA addition after 12 h storing

**Fig. 4:**
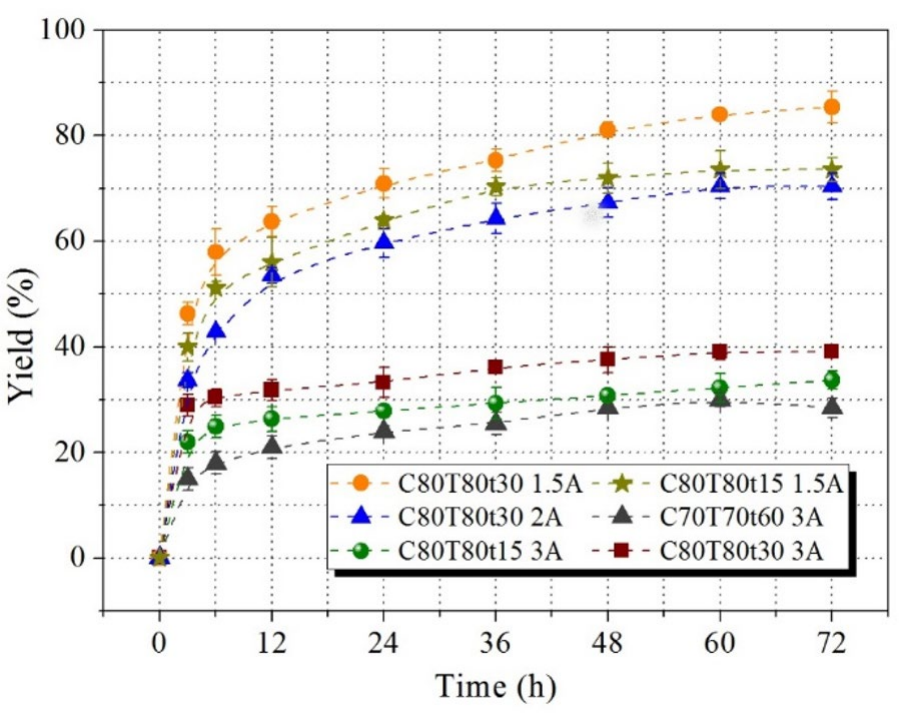
Enzymatic saccharification of substrates with different FA additions

The enzymatic saccharification of the pretreated substrates with different conditions is shown in Fig. 2b. Increasing pretreatment severity from C60T70t60 to C80T80t30, enzymatic saccharification yield increased from 34.7 ± 2.6% to 94.8 ± 1.9% after 72 h. At C80T80t15, only 4.91 ± 0.35% hemicellulose and 8.98 ± 0.58% lignin were found in the pretreated solid. The enzymatic hydrolysis efficiency of cellulose reached to 84.6 ± 0.8% after 72 h. After the partial removal of lignin and hemicellulose, pores and gaps were formed on the cell wall (Fig. 2c). The surface area was also increased from 8.81 m^2^/g (raw material) to 13.25 m^2^/g (the pretreated substrate obtained from C80T80t30). Therefore, the chemical accessibility of glucan significantly improved resulting in high enzymatic saccharification of the pretreated substrates. Although high pretreatment severity enhanced the enzyme digestibility of glucan, the native chemical structure of lignin was destroyed as revealed by a dark color (Fig. 3). We tried to use FA as a protecting agent to stabilize lignin during pretreatment. When adding FA in *p*-TsOH solution, the extracted lignin exhibited a lighter color than that of the obtained lignin without FA addition, as shown in Fig. 3. It may be the reason that the formed C–C bonds from the condensation reaction of lignin during pretreatment process cause the color to be darker under acid or high temperature conditions. Adding FA during pretreatment blocked the condensation reaction between lignin molecules resulting in not only a light apparent morphology but also the improvement of enzymatic hydrolysis of glucan. Lignin condensation causes non-productive binding with enzyme. Except for lignin backbone disruption and fractionation, the condensation/re-polymerization usually occurred during pretreatment. Aliphatic OHs elimination and condensation of lignin segments raised hydrophobicity accompanying with *p*-hydroxyphenyl re-polymerization onto lignin skeleton. More condensation involved more branched lignin sections with hydrophobic moieties and this meant that more free enzyme was trapped in complex lignin net structures. Moreover, cellulase usually contains tryptophane, phenylalanine and tyrosine which belong to hydrophobic residues. These aromatic amino acids are responsible for driving cellulase to align with pyranose rings on glucose chains through stacking and hydrophobic interactions. Unfortunately, this type of hydrophobic interaction also occurs in non-productive adsorption of cellulase onto condensed lignin. (Song et al. 2020). Meanwhile, we also investigated the effect of FA on the enzymatic saccharification of glucan. When adding 3% FA in *p*-TsOH solution, the enzymatic hydrolysis efficiency of the obtained cellulose-rich substrate was significantly reduced, as shown in Fig. 4. FA not only reacted with lignin to block new C–C bonds formation during pretreatment, but also reacted with monosaccharides and polysaccharides. In addition, it is likely that the formed 1, 3-dioxane structures from the reaction between FA and hydroxyl on cellulose surface lead to a much lower enzyme digestibility of the pretreated substrates. It has been reported that the changes in the chemical properties of cellulose surface can severely affect enzyme binding and complexation to the surface resulting in decrease of enzymatic digestibility of cellulose (Pan et al. 2006; Shuai et al. 2016). Therefore, a low concentration of FA was suggested to be used during pretreatment. When the FA addition decreased to 1.5%, the enzymatic hydrolysis of glucan reached to 85.5 ± 3.0% at 72 h. Therefore, an optimal condition (C80T80t30 with 1.5% FA) was selected for the pretreatment.

Subsequently, FT-IR spectroscopy, TGA, 2D HSQC NMR spectroscopy, and GPC were used to investigate the effects of adding FA on the structures and properties of the EL and CEL.

### Lignin characterization

**Fig. 5:**
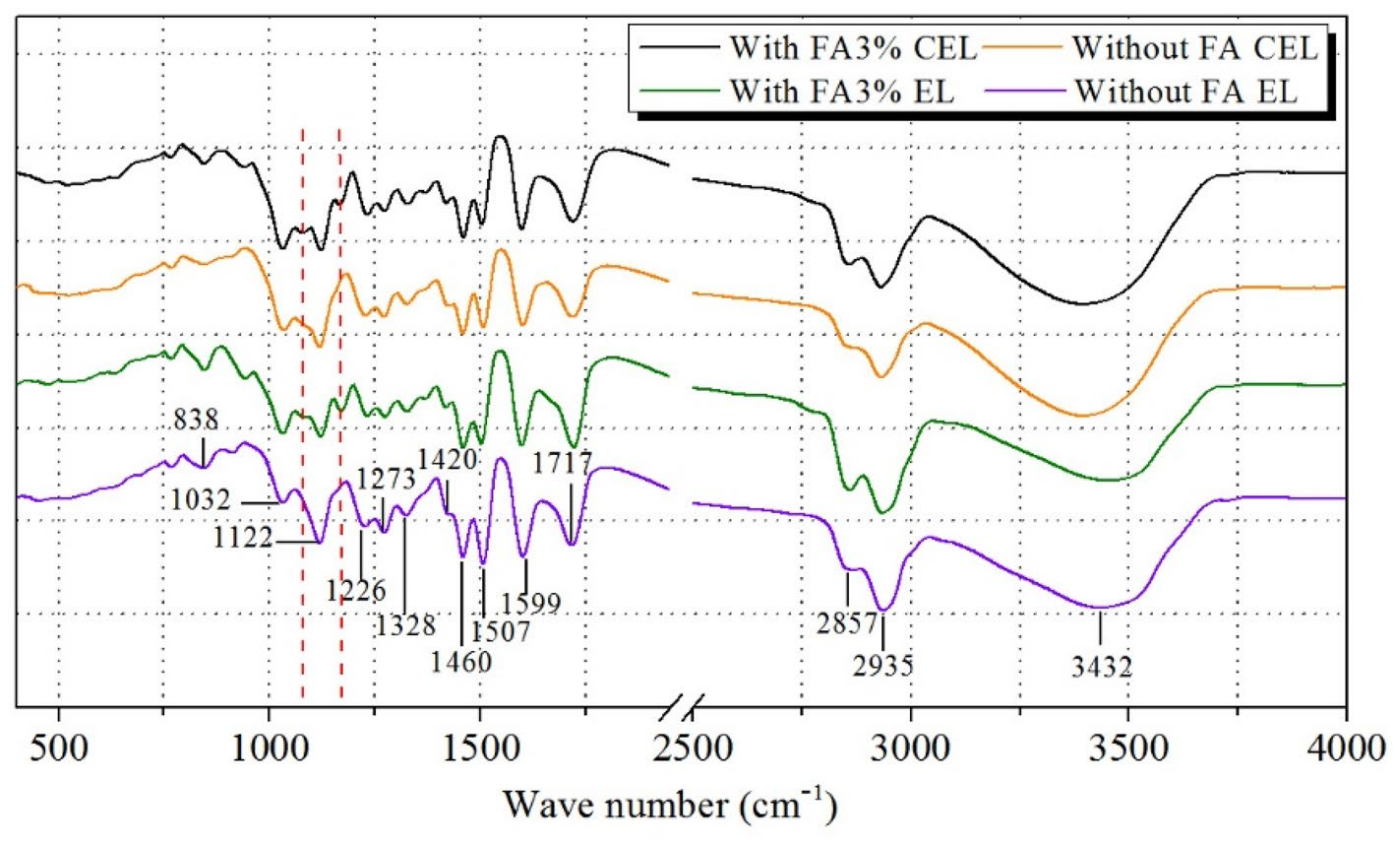
FT-IR spectra of the obtained lignin

Figure 5 shows the FT-IR spectra of the obtained lignin samples with and without FA addition, and the signals were assigned according to a previous literature (Chong et al. 2018). The spectrum of these four lignin samples showed a wide absorption band at about 3432 cm^−1^, which is related to the stretching vibration of phenolic or aliphatic OH groups. The bands at about 2935 cm^−1^ and 2857 cm^−1^ are attributed to the C–H asymmetric and symmetrical vibrations of the methyl and methylene groups, separately. The signals at 1717 cm^−1^ are assigned to the C=O stretching vibrations in conjugated carboxylic acid and ketone groups, which is more pronounced after adding FA than that without FA, suggesting that the FA stabilized lignin obtained contained more conjugated C=O group. In addition, the signals at 1599 cm^−1^ and 1570 cm^−1^ are corresponded to aromatic skeletal vibrations and the C–H deformation vibrations, respectively. The bands at 1460 cm^−1^ and 1420 cm^−1^ originated from the C–H deformations asymmetric in –CH_3_ of methoxy groups. The typical peaks of GS type lignin at 1328 cm^−1^, 1273 cm^−1^, and 1122 cm^−1^ are corresponded to the breathing vibration of syringyl (S), condensed guaiacyl (G), and guaiacyl ring breathing with C=O stretching vibration. Furthermore, the signal at 1226 cm^−1^ is ascribed to the C–C, C–O and C=O stretching. In addition, the peaks at 1032 cm^−1^ and 838 cm^−1^ are attributed to the aromatic C–H in plane deformation vibrations and the C–H out of plane stretching vibrations. In Fig. 5, similar signal profile indicated that these lignin samples obtained from different pretreatment conditions maintained their core chemical structures as that of native lignin. For the pretreatment with FA addition, new signals appeared at 1032–1122 cm^−1^ and 1122–1226 cm^−1^ in the infrared spectrum of the extracted lignin. The peak at 1032–1122 cm^−1^ represents a characteristic absorption of acetal, which is generated from the reaction between FA and lignin.

**Fig. 6:**
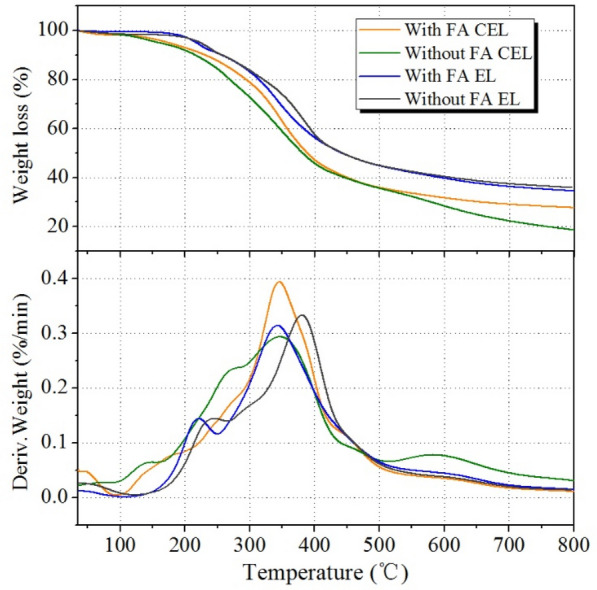
TGA curves of lignin with and without FA

The TGA curves of lignin samples obtained from the pretreatment with and without FA addition are shown in Fig. 6. Four samples showed different DTG profile below 100 °C, which was mainly caused by the removal of free water on the surface of lignin. In the range of 100–180 °C, the molecular structure of lignin was relatively stable as revealed by the horizontal TG curve. As the temperature increased to 200 °C above, the increased weight loss rate indicated that thermal decomposition reaction of lignin occurred resulting in cleavage of arylalkyl ether bonds. When temperature reached to 300–400 °C, the maximum weight loss rate was observed, which was mainly caused by oxidation or carbonylation of aliphatic hydroxyl groups and the cleavage of benzene ring and C–C bonds. When temperature risen above 500 °C, the curve was gentle, and the weight loss rate was gradually reduced, which was the finishing stage of pyrolysis. By comparison, CEL has a higher content of β-O-4 bonds, so the thermal decomposition rate is higher than that of EL. Compared with the sample without FA stabilization, the FA stabilized lignin has more residual mass at 800 °C, indicating that the lignin after acetalization has better thermal stability.

**Fig. 7:**
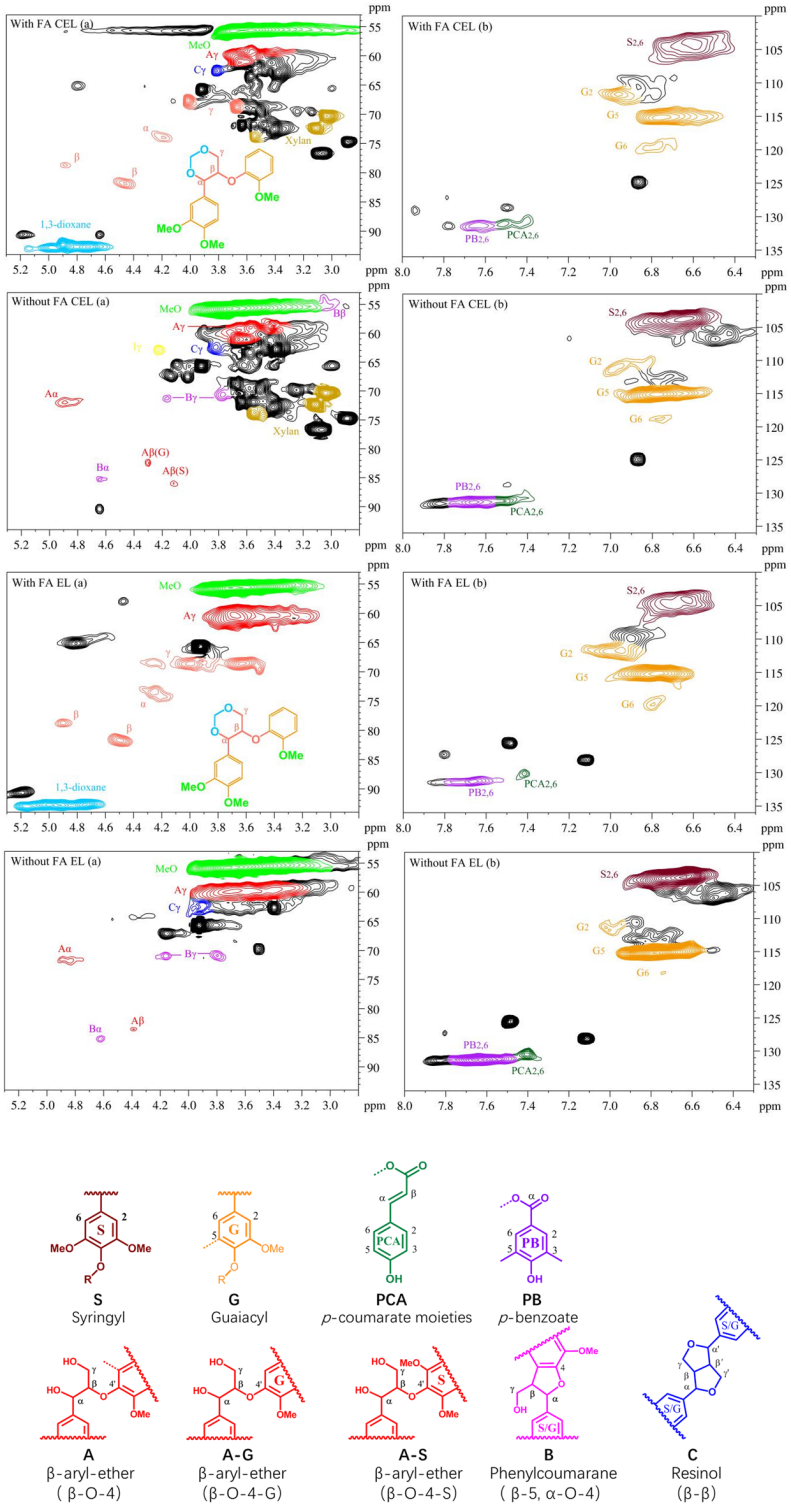
Side-chain (left) and aromatic regions (right) in the 2D HSQC NMR spectra of CEL and EL obtained from different pretreatment conditions and their main structure

We analyzed the chemical properties of CEL and EL obtained from pretreatment with and without FA addition using 2D HSQC NMR spectroscopy. Figure 7 shows the NMR spectrum concluding a side-chain region (*δ*_C_/*δ*_H_ 50.0–90.0/2.90–5.70) and an aromatic region (*δ*_C_/*δ*_H_ 100.0–135.0/6.20–7.90) along with the identified chemical structures. The assignments of main cross-signals in the spectra of lignin were assigned according to a previous publication (He et al. 2017; Ji et al. 2017; Li et al. 2019). The formation of the six-membered 1,3-dioxane structures in the presence of FA was confirmed by the 2D HSQC NMR. Typically, the signals of methoxy groups and major linkages, such as β-aryl-ether (β-O-4′, A unit), resinol (β–β′, B unit), and phenylcoumaran (β-5′, C unit) substructures, were found in the side-chain region (Fig. 7). The methoxy groups (-OCH_3_) were identified according to the cross signals at *δ*_C_/*δ*_H_ 55.60–3.71. The cross signals at *δ*_C_/*δ*_H_ 71.90/4.85, *δ*_C_/*δ*_H_ 84.40/4.40, *δ*_C_/*δ*_H_ 85.60/4.20, and δ_C_/δ_H_ 59.40/3.70 were attributed to *C*_*α*_–*H*_*α*_, *C*_*β*_–*H*_*β*_ and *C*_*γ*_–*H*_*γ*_ correlation in β-O-4′ substructure (A unit), respectively. Moreover, the *β*–*β*’ resinol substructures (B unit) were observed by the cross signals at δ_C_/δ_H_ 87.70/5.50 (*C*_*α*_–*H*_*α*_) and *δ*_C_/*δ*_H_ 63.40/3.60 (C_γ_–H_γ_). Furthermore, the β-5’ phenylcoumaran substructures (C unit) were also discovered based on the signals of C_α_–H_α_ (δ_C_/δ_H_ 85.50/4.60) and *C*_*γ*_–*H*_*γ*_ (*δ*_C_/*δ*_H_ 71.20/4.20 and 3.80). Meanwhile, the formation of the six-membered 1, 3-dioxane structures in lignin also confirmed by the signal at δ_C_/δ_H_ 92.50/4.80–5.10 in the side chain region, which indicated that FA reacted with lignin during pretreatment with *p*-TsOH hydrotrope. The disappearance of some structural signals (A, B unit) and the appearance of 1, 3-dioxane structure proved that FA addition during the pretreatment process block the reactive benzylic positions and effectively prevented the irreversible condensation during lignin extraction.

In the aromatic regions, the typical cross signals from syringyl (S) and guaiacyl (G) units were easily discovered in Fig. 7. The S unit was observed with correlation of C_2,6_–H_2,6_ at δ_C_/δ_H_ 103.80/6.69. Besides, the G units were found according to the signals at δ_C_/δ_H_ 111.10/6.98 (C_2_–H_2_), δ_C_/δ_H_ 114.70/6.71 (C_5_–H_5_), and δ_C_/δ_H_ 118.90/6.80 (C_6_–H_6_). In addition, the *p*-Coumarate acid was also identified with C_2,6_–H_2,6_ correlations at δ_C_/δ_H_ 129.80/7.50. The *p*-benzoate (PB unit) was discovered according the cross signals at C_2,6_–H_2,6_ (δ_C_/δ_H_ 131.60/7.70). In short, these signals indicated that the CEL and EL contained typical lignin structural features. Overall, adding FA in *p*-TsOH hydrotrope can block the active reaction sites of aromatic rings and hinder the formation of C–C linkages, thereby reducing the lignin condensation.

To explore the influence of FA on the molecular weight of lignin, the weight average molecular weight (*M*_w_), number average molecular weight (*M*_n_) and polydispersity index (PDI, *M*_w_/*M*_n_) were determined by GPC and the results are shown in Table 1. The FA stabilized lignin showed a higher molecular weight (5371 g/mol) and lower polydispersity index (PDI 1.63) than that of extracted lignin without FA reaction. The addition of FA promoted the acetal reaction between FA and lignin resulting in a high molecular weight. The low polydispersity index (PDI < 2) indicates that the obtained lignin is a uniform lignin fragment with relatively stable properties and high industrial value.

**Table 1:** Weight-average molecular weight, number-average molecular weight (M_n_) and polydispersity index (PDI, *M*_w_/*M*_n_) of the lignin with FA and without FA

Samples	*M*_w_ (g/mol)	*M*_n_ (g/mol)	*M*_w_*/*M_n_
With FA CEL	5371	3285	1.63
Without FA CEL	4412	2590	1.70
With FA EL	4558	2875	1.58
Without FA EL	4057	2489	1.63

### High titer ethanol production via a Q-SSF process

**Fig. 8:**
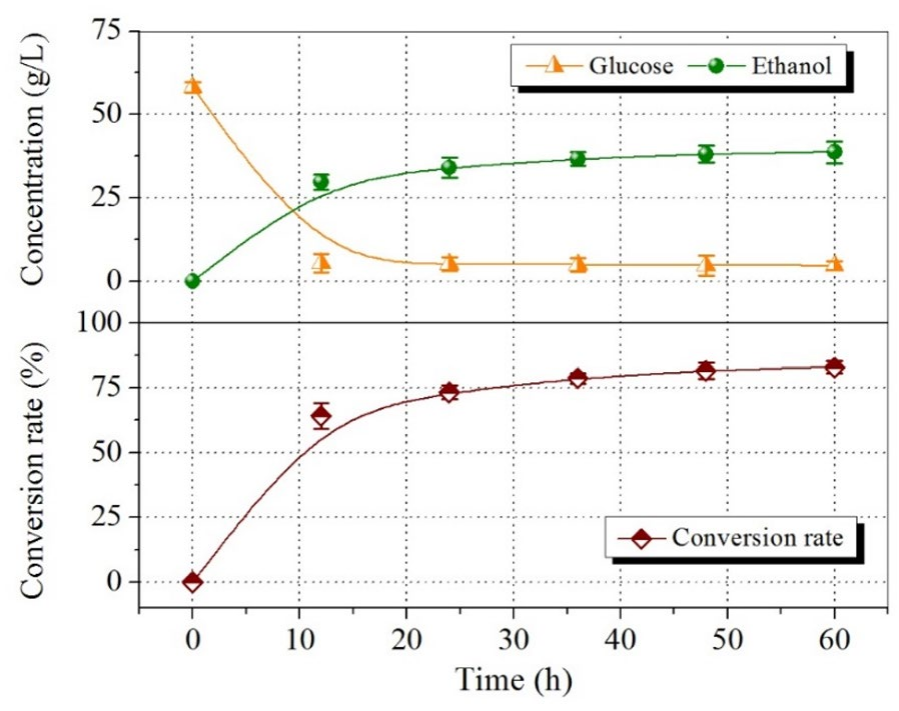
Concentrations of glucose and ethanol and the ethanol yield in the Q-SSF process

**Fig. 9:**
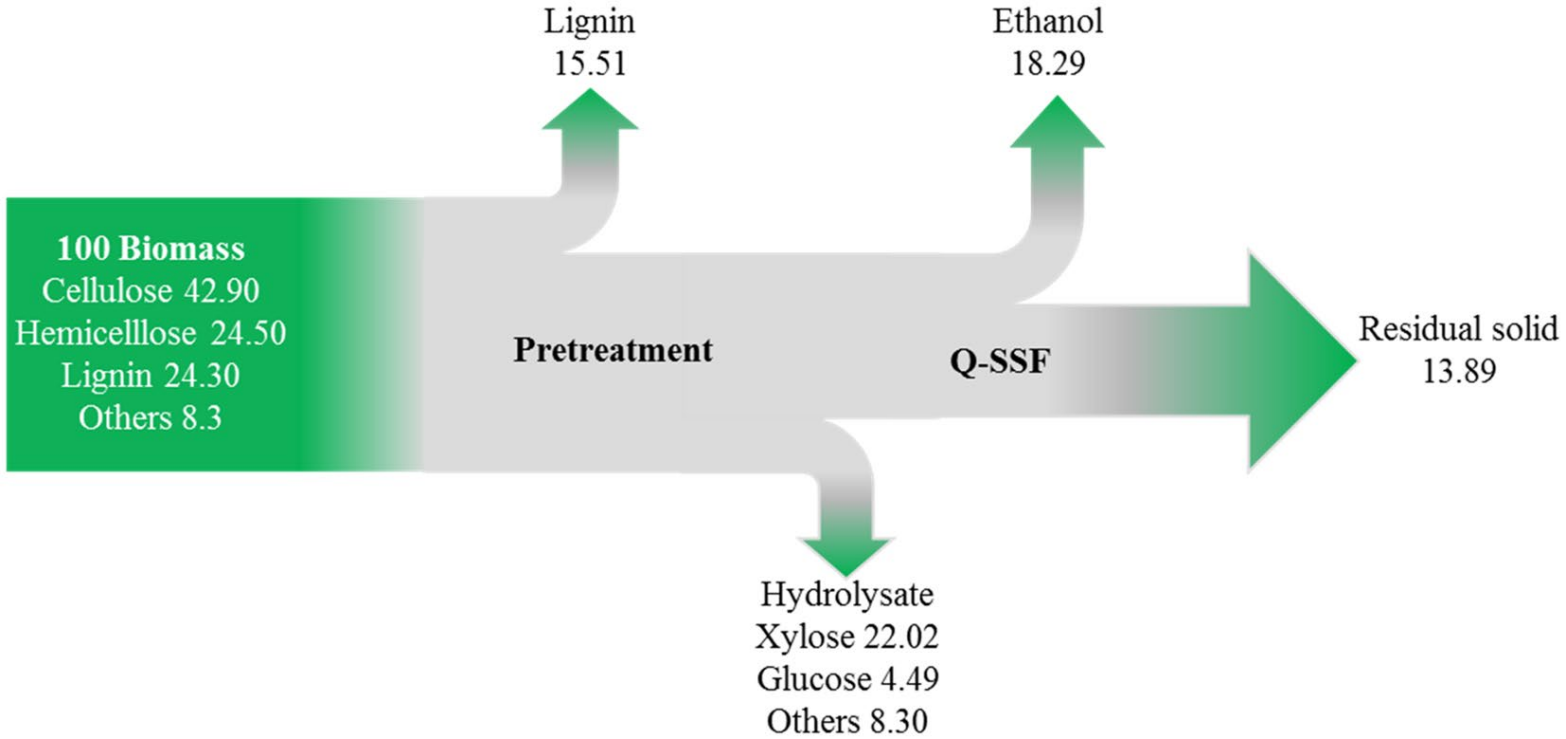
Mass balance of biomass via hydrotrope pretreatment and Q-SSF

Because FA addition of 1.5% showed a negligible effect on enzymatic saccharification of glucan, the final glucan-rich solids obtained from C80T80t30 with 1.5% FA were converted into ethanol by a Q-SSF process. The changes of glucose and ethanol concentrations and ethanol conversion rate during Q-SSF are shown in Fig. 8. Within 12 h, the glucose concentration dropped rapidly and the ethanol concentration reached to 29.80 ± 2.3 g/L after 12 h of yeast inoculation. After 60 h of fermentation, the ethanol concentration reached a maximum value of 38.70 ± 3.3 g/L, which was equivalent to the theoretical ethanol yield of 82.90 ± 2.2%, while the concentration of residual glucose was only 4.69 ± 1.4 g/L. It can be seen that there was no obvious inhibitory effect during the process of converting glucose to ethanol, indicating that the pretreatment procedure, specifically, using a recyclable *p*-TsOH hydrotrope to dissolve lignin under mild condition and stabilized lignin with FA as a quenching agent during pretreatment, achieved both high-quality lignin extraction and efficient enzymatic saccharification of glucan for high-titer ethanol production. The mass balance of biomass is shown in Fig. 9. Therefore, this study is important to the valorization of lignocellulosic biomass.

## Conclusions

In this study, we used a recyclable *p*-TsOH hydrotrope to pretreat biomass under mild condition and stabilized lignin with FA as a quenching agent during pretreatment. At C80T80t30, the removal of hemicellulose and lignin achieved 83.8 ± 0.7% and 67.1 ± 2.6%, respectively. Meanwhile, FA can effectively prevent the vicious condensation of lignin as revealed by a lighter color. The lignin samples were characterized by FTIR spectroscopy, TGA, 2D HSQC NMR spectroscopy, and GPC. The results indicated that the FA stabilized lignin showed a higher molecular weight (5371 g/mol) and polydispersity index (PDI 1.63). Subsequently, the pretreated solids obtained from C80T80t30 with 1.5% FA addition were converted into ethanol by a Q-SSF process. The maximum ethanol concentration reached 38.7 ± 3.3 g/L, which was equivalent to the theoretical ethanol yield of 82.9 ± 2.2%. In summary, the pretreatment strategy achieved both high-quality lignin extraction and efficient enzymatic saccharification of glucan for high-titer ethanol production.

## Data Availability

The data sets used and/or analyzed during the current study are available from the corresponding author on reasonable request.

## Abbreviations

*p*-TsOH: *p*-Toluene sulfonic acid
FA: Formaldehyde
Mw: Molecular weight
Q-SSF: Quasi-simultaneous Saccharification and Fermentation Process
AFEX: Ammonia Fiber Explosion
EL: Extracted Lignin
CEL: Cellulolytic Enzyme Lignin
TGA: Thermogravimetric Analysis
GPC: Gel Permeation Chromatography
PDI: Polydispersity Index
